# Through the Long COVID Looking-Glass: An Unremitting Conundrum of Mitochondria?

**DOI:** 10.3390/ijms27219753

**Published:** 2026-10-30

**Authors:** Svetlana Kononova, Natalia Bobkova, Sergey Leonov, Gennadiy Sukhikh

**Affiliations:** 1 Institute of Cell Biophysics, Russian Academy of Sciences, Federal Research Center, Moscow Region, Puschino 142290, Russiasergey.leonov@yahoo.com (S.L.); 2 Institute of Future Biophysics, Moscow Center for Advanced Studies, Moscow 123592, Russia; 3 National Medical Research Center for Obstetrics, Gynecology and Perinatology Named After Academician V.I. Kulakov, Moscow 117997, Russia

**Keywords:** long COVID, mitochondria, RAAS, p53, proteins of SARS-CoV-2, neurodegeneration

## Abstract

Plentiful data indicate a high post-COVID risk of developing pathologies that are long-term and result from disruption of multiple cellular processes. The significance of this data becomes increasingly clear as it links the pandemic’s effects to enduring health problems which could affect millions. These conditions are usually caused by different problems with mitochondria. These problems can include less efficient energy production and increased removal of damaged cells, showing how complex cell health is. Having other health problems such as high blood pressure, type 2 diabetes, brain diseases, and cancer related to renin–angiotensin–aldosterone system (RAAS) imbalance worsened COVID-19 and its lasting effects. This highlights the importance of focused treatments for these at-risk groups. This review seeks to evaluate the information on this issue, carefully studying the features and symptoms often associated with mitochondrial disorders. What happens next in the pandemic is still to be seen, but we know there is much to learn. It is important to lessen the effects of the pandemic on people with comorbidities as much as possible.

## 1. Introduction

The recent coronavirus pandemic (COVID-19), caused by the SARS-CoV-2 virus (severe acute respiratory syndrome coronavirus 2), has resulted in nearly 780 million cases and over 7.1 million deaths, according to the World Health Organization, 2026 [1]. But if during the COVID-19 pandemic the question of choosing a treatment strategy was initially acute, then later the question arose about the risk of developing long-term consequences, including neurodegenerative and oncological diseases [2,3,4,5] in people who had COVID-19. SARS-CoV-2 infection promoted the development of chronic inflammation, mitochondrial dysfunction, oxidative stress, immune dysregulation, cellular senescence, and impaired apoptosis and autophagy [4]. Numerous data indicate a high post-COVID risk of developing pathologies that are long-term and result from disruption of multiple cellular processes. These pathologies are typically underpinned by a variety of mitochondrial dysfunction manifestations, ranging from decreased oxidative phosphorylation to activation of mitophagy and autophagy [4].

Therefore, in many reviews devoted to the mechanisms of COVID infection development, mitochondria, present in the overwhelming majority of cells in the body, are considered one of the main targets of both direct and indirect action of the SARS-CoV-2 virus, which uses mitochondria for its replication [6,7,8,9]. Multiorgan damage in patients with COVID-19 was also facilitated by the tropism of this virus for the membrane form of angiotensin-converting enzyme 2 (ACE2), which is expressed in a wide range of different tissues and organs and is an essential receptor for virus entry into the cell [10,11,12,13,14,15]. It is important to note that ACE2 belongs to the counter-regulatory branch of the renin–angiotensin–aldosterone system (RAAS), which is responsible for maintaining homeostasis in the body [16]. Perhaps this is why the presence of comorbidities such as hypertension, type 2 diabetes, neurodegenerative diseases, and oncological diseases, which are characterized by RAAS imbalance [17,18,19,20,21], along with mitochondrial disorders, significantly exacerbated the severity of COVID-19 and its long-term consequences. It is still unclear what changes at the molecular and cell level in the early phase of the disease could indicate a bad outlook for serious issues later. This poses a big challenge for both practical medicine and basic science. This review aims to assess the available data on this subject, with a focus on the traits of mitochondrial disorders.

## 2. ACE2 as a Component of the RAAS and SARS-CoV-2 Infection

ACE1 and ACE2 are key enzymes in the RAAS, regulating its opposing axes. One of the main products of ACE2 is angiotensin (Ang) (1–7), which is produced from Ang-II, a product of a reaction catalyzed by ACE1. An imbalance between ACE2 and ACE1 activity leads to a cascade of biological and pathological consequences [22].

The *ACE2* gene, located on the X chromosome (Xp22.2), can express several RNA transcript variants as a result of alternative splicing [23,24]. The major isoform 1 is a type 1 transmembrane glycoprotein with a catalytic center in the extracellular domain, which can function not only as an enzyme but also as a component of the heterotetrameric transporter complexes B^0^AT1, B^0^AT3, and SIT1, encoded by the genes *SLC6A19*, *SLC6A18*, and *SLC6A20*, respectively. In addition, it can be a part of a number of supramolecular cellular complexes. All this together determines the involvement of ACE2 in various cellular processes [23].

Inactivation of the membrane-bound form of ACE2 (mACE2) following SARS-CoV-2 infection resulted in suppression of the ACE2/Ang (1–7)/mitochondrial assembly receptor (MasR) axis in cells, increased Ang II levels, and provoked RAAS imbalance [21]. This triggered pathophysiological processes that determined the spectrum of clinical manifestations of COVID-19, as increased Ang II levels can contribute to inflammation and oxidative stress [25,26].

ACE2 function is associated with the activation of the phosphoinositide 3-kinase (PI3K)/Protein Kinase B (AKT), Ras/Mitogen-activated protein kinase (MAPK), and Tuberous Sclerosis Complex (TSC)/mechanistic Target of Rapamycin (mTOR), Receptor Tyrosine Kinase (RTK) pathways and with the inactivation of the cell cycle, DNA damage, epithelial–mesenchymal transition (EMT), and the androgen receptor, which it inhibited more often than activated (12% inhibition vs. 7% activation) [27]. RAAS imbalance causes activation of the MAPK/extracellular signal-regulated kinase (ERK) pathways, which promotes Transforming Growth Factor-β (TGF-β) production [3,28].

## 3. Mitochondria and SARS-CoV-2 Infection

Mitochondria are involved in a wide range of metabolic and signaling pathways that regulate critical cellular functions, including adenosine 5′-triphosphate (ATP) synthesis, production and regulation of reactive oxygen species (ROS), inflammation, lipid metabolism, maintenance of cellular calcium and iron homeostasis, cell death (apoptosis, necrosis, and pyroptosis), as well as regulation of autophagy and regulation of the synthesis of steroid hormones such as cortisol and estrogen [7,9].

Mitochondria play a vital role by producing ATP through a process called oxidative phosphorylation (OXPHOS). This process involves the electron transport chain (ETC) and ATP synthase, located on the inner mitochondrial membrane. Eleven sites of the ETC, where superoxide and hydrogen peroxide are formed, make mitochondria an important source of cellular ROS. Normally, a controlled pool of mitochondrial ROS (mt-ROS) functions as signaling molecules necessary for the maintenance of numerous processes and signaling cascades, but uncontrolled increased production of mt-ROS leads to oxidative stress, causing cell damage [29,30].

Mitochondria of heterogeneous populations of immune cells play a crucial role in activating innate immunity. Mitochondrial antiviral signaling protein (MAVS), located on the outer mitochondrial membrane, is part of the signalosome complex. Under uninfected conditions in the body, MAVS interaction with mitofusin-2 (Mfn2) prevents the antiviral response by blocking MAVS’s link to the retinoic acid-inducible gene I (RIG-I) receptor.

During a viral infection, RIG-I finds foreign RNA. This activates MAVS, starting the antiviral signaling that leads to the production of interferon (IFN) α/IFNβ. This happens by activating nuclear factor-κB (NF-κB) and interferon regulatory factor 3 (IRF3). NF-κB activation is also important for initiating adaptive immunity [7,8,9]. Other mitochondrial outer membrane proteins include the voltage-dependent anion channel (VDAC) and the antiapoptotic proteins Myeloid cell leukemia-1 (Mcl-1) and B-cell lymphoma 2 (Bcl-2) [31].

The amount of circular mitochondrial DNA (mtDNA) in mitochondria varies depending on the cell type. Human heart and brain cells have the most copies of mtDNA. The frontal and cingulate cortex have about twice as many copies as the hippocampus and hypothalamic parts. High copy number confers resistance to lipid peroxidation and ferroptosis upon loss of glutathione phospholipid hydroperoxidase [32].

**Figure 1:**
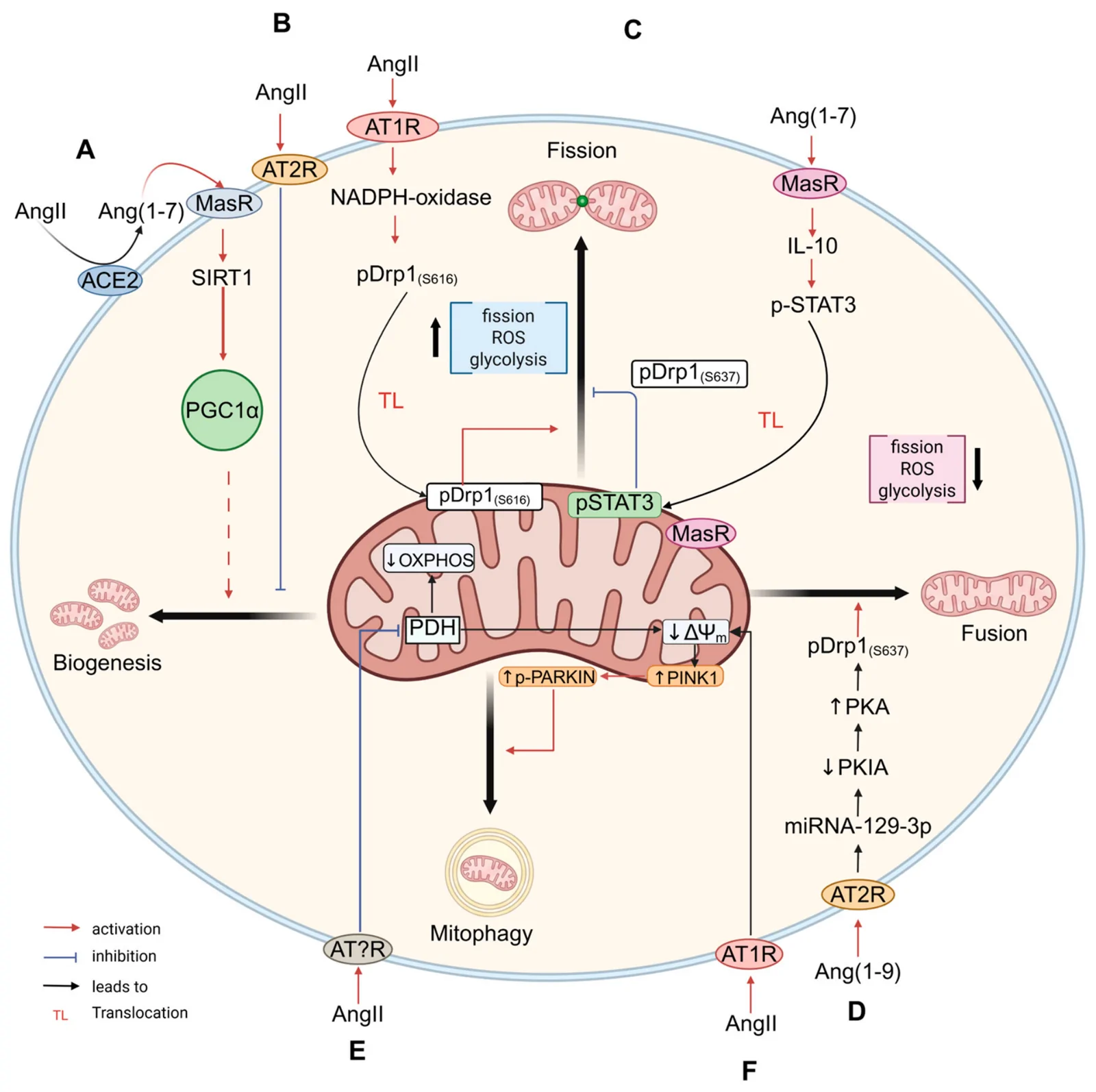
The interaction of the renin–angiotensin–aldosterone system (RAAS) and mitochondria. (**A**) The activation ACE2/MasR/SIRT1/PGC-1α axis helps mitochondrial biogenesis [36,37]. (**B**) The interaction of Ang II with AT2R inhibits mitochondrial biogenesis in C2C12 mouse muscle cells [38]. (**C**) In dopaminergic neurons Ang II promotes mitochondrial fission by activating the Ang II/AT1R/NADPH oxidase axis, which promotes phosphorylation of Drp1 at Ser616 and its translocation to mitochondria. Ang (1–7) counteracts the microglial inflammatory response. Its effect is mediated by IL-10, specifically by decreasing the post-transcriptional phosphorylated Drp1 at Ser637 and translocation of STAT3 to mitochondria where activation of the mitochondrial Ang (1–7)/MasR axis increased the phosphorylated form of STAT3 at Ser727 [39]. (**D**) The interaction of Ang (1–9) with AT2R regulates mitochondrial fusion and Ca^2+^ uptake capacity through ER-mitochondria contact sites via the AT2R/miR-129-3p/PKA signaling pathway in cardiomyocytes [40]. (**F**) In cardiomyocytes Ang II binding to AT1R decreases ΔΨm, which prevents PINK1 degradation, increasing levels of total and phosphorylated PINK1, which promotes Parkin translocation and phosphorylation and enhances mitophagy [41]. (**E**) Ang II promotes blocking the PDH, which could lower OXPHOS and change ΔΨm in rat colon cells [42]. ACE2, angiotensin-converting enzyme 2. MasR, mitochondrial assembly receptor. SIRT1, sirtuin I. PGC-1α, peroxisome proliferator-activated receptor gamma coactivator 1-alpha. Drp1, Dynamin-related protein 1. Ang, angiotensin. AT1R, Angiotensin II type 1 receptor. AT2R, Angiotensin II type 2 receptor. STAT3, Signal Transducer and Activator of Transcription 3. PKA, protein kinase A. PINK1, PTEN-induced kinase 1. PDH, pyruvate dehydrogenase complex. ΔΨm, mitochondrial membrane potential. OXPHOS, oxidative phosphorylation. Created in BioRender. Nofal, Z. (2026) https://BioRender.com/4u8qvhg.

Importantly, mitochondria are intracellular components of the RAAS. It has been shown that Angiotensin II type 1 receptor (AT1R), Angiotensin II type 2 receptor (AT2R), MASR, and Mas-related G-protein coupled receptor member E (MrgE) receptors [33,34,35] are localized on the inner mitochondrial membrane. Moreover, the presence of renin in them allows them to be considered an autonomous local RAS [34]. Through the ACE2/Ang (1–7)/MasR axis, ACE2 can influence the functioning of mitochondria and their content in the cell (Figure 1).

Ang II lowers the number of mitochondria in C2C12 mouse muscle cells by suppressing genes for mitochondrial biogenesis. This happens when it interacts with AT2R, not AT1R. Furthermore, Ang II increased mt-ROS production and decreased mitochondrial membrane potential (ΔΨm). These effects of Ang II were significantly suppressed by blockade of both AT1R and AT2R. Chronic administration of Ang II to mice also decreased muscle mitochondrial content [38]. In dopaminergic neurons and microglia, Ang II promoted mitochondrial fission by activating the Ang II/AT1R/NADPH oxidase axis, which promoted phosphorylation of Dynamin-related protein 1 (Drp1) at Ser616 and its translocation to mitochondria [39]. Ang II raised the level of pyruvate dehydrogenase kinase 2 (PDK2) in rat colon cells. This may help block the pyruvate dehydrogenase complex (PDH), which could lower OXPHOS and change ΔΨm and lipid balance [42]. Furthermore, Ang II treatment of cardiomyocytes resulted in decreased ΔΨm, which prevented PTEN-induced kinase 1 (PINK1) degradation, resulting in increased levels of total and phosphorylated PINK1, which promoted Parkin translocation and phosphorylation and enhanced mitophagy [41]. Diminazene aceturate (DIZE) activates ACE2, which helps mitochondrial biogenesis through MasR and SIRT1. SIRT1 increases the expression of peroxisome proliferator-activated receptor gamma coactivator 1-alpha (PGC-1α), a key regulator for mitochondria [36,37]. In *ACE2* gene knockout mice, mRNA levels of *PGC-1α*, *PPARα*, *PPARγ*, *CPT-1α*, and *MCAD* genes were reduced [43]. Another ACE2 product, Ang (1–9), derived from Ang I and interacting with AT2R, regulates mitochondrial morphology and Ca^2+^ uptake capacity through ER-mitochondria contact sites via the AT2R/miR-129-3p/protein kinase A (PKA) signaling pathway in cardiomyocytes. AT2R activation leads to increased expression of miR-129-3p, which reduces the level of PKIA (protein kinase A inhibitor), leading to the activation of PKA, which phosphorylates DRP1 and ultimately promotes mitochondrial fusion [40]. Phosphorylation at various residues of DRP1 plays an important role in the regulation of mitochondrial fission [44].

Thus, these data suggest that disruption of mACE2 function during SARS-CoV-2 infection might also be involved in mitochondrial dysfunction. But the main point is that the virus uses mitochondria for its own replication [6,7,8,9].

The SARS-CoV-2 virus contains positive-sense single-stranded RNA (+ss RNA) as its genomic RNA (gRNA). It has a nucleocapsid (N-) protein that interacts with this RNA, plus Spike (S-), membrane (M-), and envelope (E-) proteins. The presence of multiple open reading frames (ORFs) in the genome also allows encoding non-structural (*ORF1a* encodes NSP1-NSP11, *ORF1b*-NSP12-NSP16) and accessory proteins (expressed from *ORFs 3a*, *3b*, *3c*, *6*, *7a*, *7b*, *8*, *9b*, *9c* and *10*) [6,8,9,45]. Its life cycle includes cell entry, translation, replication, assembly, and exit from the infected cell. After SARS-CoV-2 enters the cell and releases gRNA into the cytosol, along with *ORF1a* and *ORF1ab*, translation immediately initiates, and two polyproteins (pp1a and pp1ab) are synthesized. These polyproteins are autocleaved by papain-like protease (PLpro) (NSP3) and 3C-like protease (3CLpro) (NSP5) into 16 nonstructural proteins (NSPs), 15 of which are part of the viral replication and transcription complex (RTC) [8,46]. The RTC makes daughter gRNA molecules by producing an intermediate double-stranded RNA (dsRNA) in double-membrane vesicles (DMV) and a coiled membrane network (CM), called “viral replication organelles,” as well as in various lipid droplets. DMVs are formed by the endoplasmic reticulum (ER) and mitochondria under the influence of stress caused by SARS-CoV-2 entry [8,9]. Translation of structural proteins occurs in the cytosol [47].

In the cytosol, the virus is vulnerable to the innate immune system. Viral replication in the DMV allows the virus to both evade recognition by the host immune system and initiate mechanisms to suppress the innate immune system. The exact mechanism by which SARS-CoV-2 mitochondria are captured is unknown, but computer modeling predicts the localization of the RNA transcripts *Orf1ab*, *Orf3a*, *Orf6*, *Orf7a*, *Orf7b*, *Orf8*, *Orf10*, *S*, *E*, *M*, and *N* in the mitochondria [6,9]. All translated structural proteins and synthesized genomic copies of SARS-CoV-2 are transported to the ER and Golgi apparatus, after which self-assembly of new virions occurs, which leave the cell via exocytosis [9].

**Figure 2:**
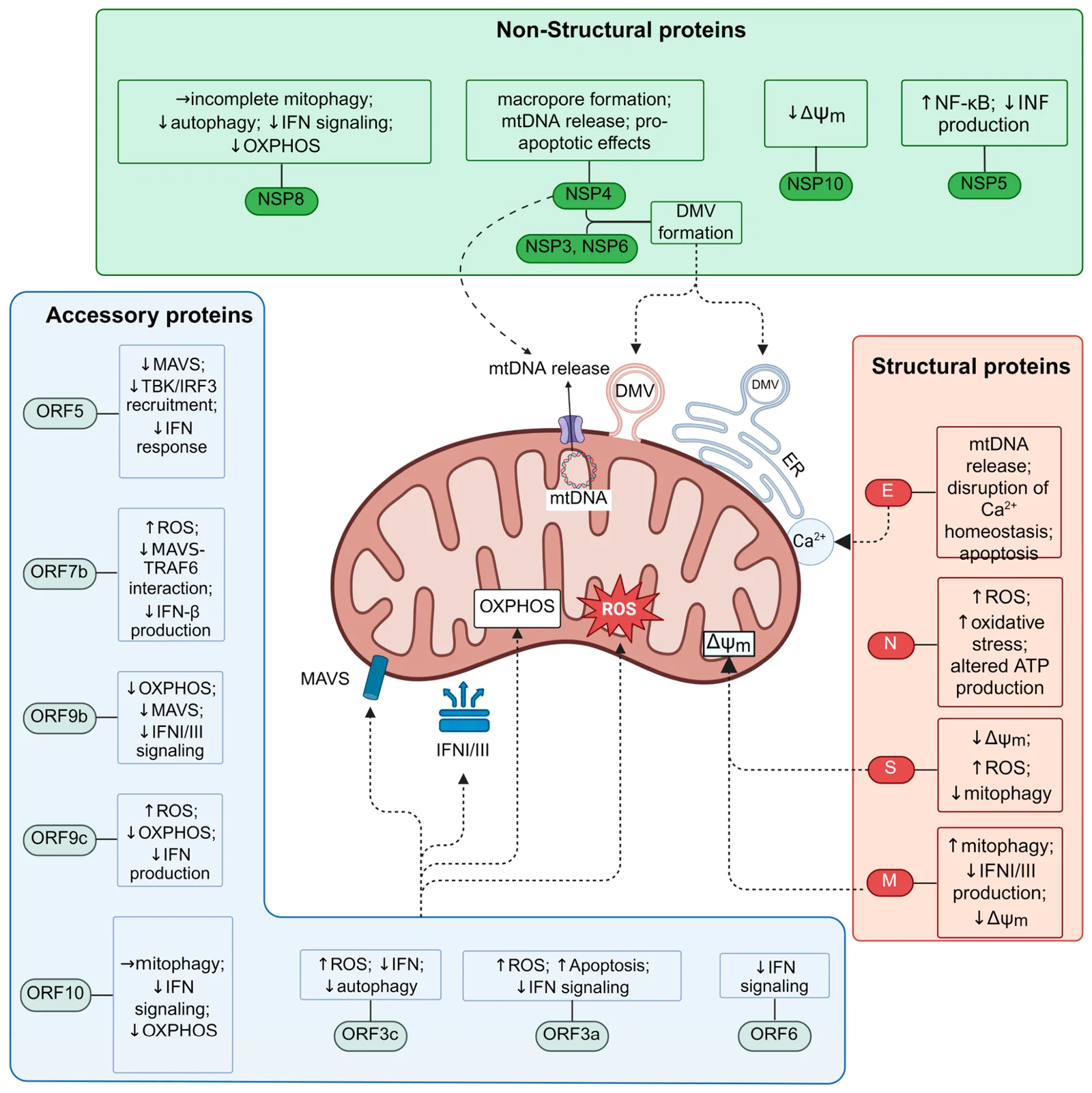
The effect of SARS-CoV-2 proteins on mitochondria. Viral proteins promote disruption of OXPHOS regulation, an increase in ROS production, suppression of IFN production, and DMV formation for viral replication. NSP, Non-structural protein. ORF, protein is translated from an open reading frame. MAVS, Mitochondrial antiviral signaling protein. IFN, interferon. IRF3, interferon regulatory factor 3. ΔΨm, mitochondrial membrane potential. OXPHOS, oxidative phosphorylation. ROS, reactive oxygen species. mtDNA, mitochondrial DNA. DMV, double-membrane vesicles. ER, endoplasmic reticulum. NF-κB, nuclear factor-κB. TBK1, TANK-binding kinase 1. → points to an incomplete process. Up and down arrows (↑/↓) indicate an increase and decrease, respectively. Dashed arrows indicate mitochondrial processes affected by SARS-CoV-2 proteins. Created in BioRender. Nofal, Z. (2026) https://BioRender.com/lrngjfu.

Mitochondrial dysfunction caused by viral mitochondrial hijacking, in addition to disruption of OXPHOS regulation and decreased ATP production, includes a failure of antioxidant defense mechanisms, an increase in the amount of ROS, and oxidative damage to molecules (Table 1, Figure 2).

SARS-CoV-2 viroporins E and Orf3a promoted Ca^2+^ influx from the ER into mitochondria, stimulating VDAC expression. This activated the tricarboxylic acid cycle (TCA) and led to the formation of excess reduced form nicotinamide adenine dinucleotide (NADH), which overloaded the ETC and increased mtROS production. This resulted in disruption of the mitochondrial membrane integrity and the release of mtDNA into the cytosol and circulation [7,29]. Circulating mtDNA fragments are damage-associated molecular patterns (DAMPs) for TLR9 on T cells and macrophages. TLR9 detects unmethylated CpG sites in them and activates NF-kβ and MAPKs, which lead to a proinflammatory response. This process begins the release of proinflammatory cytokines such as interleukin (IL)-6, IL-8, and Tumor Necrosis Factor alpha (TNF-α). TNF-α and IL-6 inhibited the mitochondrial OXPHOS pathway, causing a decrease in ATP production and increased mt-ROS production. Cellular metabolism shifted toward aerobic glycolysis, which facilitated viral replication by providing it with structural components. Aerobic glycolysis, or the “Warburg effect,” can be initiated via the NF-κB pathway by SARS-CoV-2 proteins, particularly in endothelial and immune cells [7,60,61]. Activation of mitochondrial innate immune signaling genes (*CMPK2*, *PNPT1*, *IFI27*, and *IRG1*), as shown in BALB/c and C57BL/6 mice, was initiated after the virus reached its peak load and triggered transcriptional suppression of mitochondrial genes and the induction of glycolysis [29].

A study of nasopharyngeal samples and autopsy samples from COVID-19 patients found that some genes had higher activity. Most hypoxia-inducible factor-1 alpha (HIF-1α) target genes were upregulated in lung, heart, kidney, and liver autopsy specimens, although this was less pronounced in nasopharyngeal specimens. mTOR gene activity was raised in lung and lymph node samples, slightly in nasopharyngeal samples, but greatly reduced in heart samples and somewhat in kidney and liver samples. While increased mTOR activity in nasopharyngeal and lung tissues promotes glycolysis, decreased mTOR activity in the heart, kidney, and liver may reflect a global downregulation of bioenergetic genes [29].

TNF-α was also involved in initiating the inflammatory cascade through activation of NF-κB, while IL-6 involved activation of the signal transducer and activator of transcription STAT3 [60].

In the cytosol, cGAS finds mtDNA as a danger signal. It uses cGAMP to turn on STING1, which leads to the production of IFN-I. Along with mt-ROS, mtDNA activates the NLRP3 inflammasome, which contains pyrin-domain-containing protein 3 (NLRP3) and regulates the proinflammatory cascade and pyroptosis. Once NLRP3 DAMP is active, ASC (Apoptosis-associated speck-like protein that has a CARD) gathers procaspase-1. When the inflammasome is formed, activated caspase-1 modifies Pro-IL-1B and Pro-IL-18 into their active versions, IL-1β and IL-18. In addition to these proinflammatory cytokines, activated innate immunity induces the production of prostaglandins, interleukins and chemokines, and activates the complement system, triggering an inflammatory cascade [7].

In severe COVID-19 patients, mitochondrial dysfunction caused by SARS-CoV-2 resulted in a significant increase in ROS, which contributed to a sharp rise in levels of the proinflammatory cytokines TNF-α, IL-1β, IL-6, and IL-18, known as a cytokine storm. This exacerbated the inflammatory state and promoted the development of the inflammasome response [7]. The severity of the cytokine storm, determined by the presence of preexisting metabolic dysfunction, could lead to viral clearance, multiple organ failure, and death [8]. Mitochondrial damage in severe COVID-19 was confirmed by a decrease in the level of mitochondrial proteins, including the MOTS-c peptide, VDAC-1, and humanin [9], in extracellular vesicles isolated from the blood of patients. SARS-CoV-2-induced oxidative stress caused DNA damage in all patients with COVID-19 [62,63], and the virus also damaged various components of the DNA damage response pathway [64]. The level of DNA damage in leukocytes of patients with COVID-19 was almost 2-fold higher than that in the control group, and it was higher with more severe disease, and it was more pronounced in men than in women [62,65].

Inhibition of SARS-CoV-2 p53 expression can impair DNA damage repair, disrupt tissue homeostasis, and impair the immune response [66], activating a number of signaling pathways, particularly Epidermal Growth Factor (EGF) and TGF-β [67]. p53 is maintained at low levels in most normal tissues and primarily controls the cell cycle via the cyclin G1/Mdm2/p53 axis. It is also involved in DNA repair, the regulation of apoptosis, autophagy, cellular stress responses, and cellular senescence. It can exhibit both antiviral and proviral activity, participates in the regulation of innate immune responses, including the IFN-I response, and is also involved in the regulation of the inflammatory response [46,68]. p53 regulates the expression of approximately 500 target genes [2] and is one of the transcription factors for the *ACE2* gene, regulating its expression in a tissue-specific and sex-specific manner [69]. During SARS-CoV-2 infection, the p53 signaling pathway was one of the most enriched signaling pathways [46]. A Signaling Pathway Reporter Screen of 21 SARS-CoV-2 proteins in HEK293T cells showed that, with the exception of nsp2, nsp7, nsp8, nsp12, orf7b, and orf8 proteins, they can influence the expression of reporter genes in the p53, TGFβ, c-Myc, Hypoxia, AP-1, Notch, and NF-κB signaling pathways. Among SARS-CoV-2 proteins, both inducers and repressors of signaling pathways were identified. The p53 signaling pathway could be induced by orf9b, orf9c, orf10, and the N protein, while its repressors—nsp5, nsp15, orf3a, and orf6. Notably, orf9b, orf9c, and orf10, but not the N protein, were also inducers of other signaling pathways, including NF-κB. In addition, the E and M proteins, nsp1, nsp14, orf7a, and orf3a were inducers of the NF-κB signaling pathway, and the N protein could also induce the TGFβ pathway [68]. An in silico study demonstrated the ability of the viral S2 subunit to physically interact with p53 and BRCA-1/2 proteins [70]. Overexpression of p53 significantly reduces viral production in SARS-CoV-2-infected cells [68], while SARS-CoV-2 is able to enhance MDM2 (mouse double minute 2) expression [46]. MDM2 expression can be induced by NF-κB [46]. MDM2 or HDM2 (in humans) is an E3 ubiquitin ligase that can ubiquitinate both p53 and ACE2 and induce their degradation [71]. The deubiquitinating activity of PLpro towards host MDM2 stabilized MDM2, which in turn promoted accelerated proteasomal degradation of basal p53 [46]. Inhibition of MDM2 by Nutlin-3 or RG-7112 in ACE2-expressing A549-hACE2 cells and wild-type p53 cells infected with SARS-CoV-2 effectively blocked the cell cycle and inhibited IL-6, NF-κB, and IFN-λ expression and virion release [72]. Furthermore, nsp3 is proposed to lead to the stabilization of the ubiquitin E3 ligase RCHY1 (Ring Finger and CHY Zinc Finger Domain Containing 1), which ubiquitinates P53 for subsequent degradation [25].

**Figure 3:**
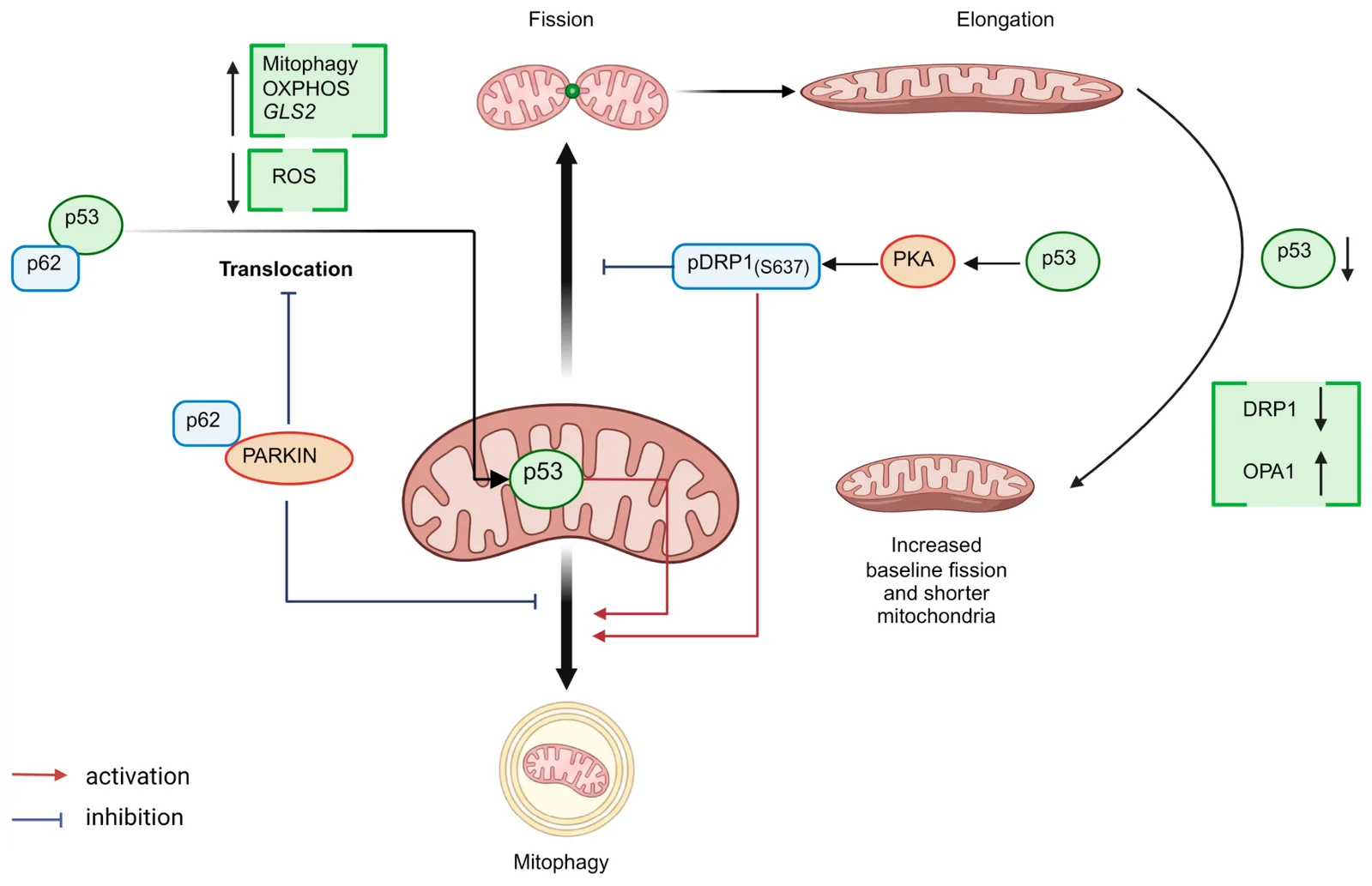
The interaction of p53 and mitochondria. The p62 protein promotes mitochondrial localization of p53 via its UBA domain [73], and p62 interaction with Parkin prevents its translocation to damaged mitochondria, inhibiting mitophagy [30]. Activation of PKA by p53 enhances inhibitory phosphorylation of DRP1 and leads to suppression of mitochondrial fission and stimulates their elongation. Suppression of p53 expression reduces mitochondrial length by altering OPA1 and DRP1 expression levels, which improves mitophagy and reduces mt-ROS levels [30]. In mitochondria, p53 raises apoptosis, defends mtDNA from mutations, and supports mtDNA copy numbers and mt-ROS homeostasis [74]. *GLS2*, glutaminase 2. Drp1, dynamin-related protein 1. PKA, protein kinase A. OPA1, optical atrophy protein 1. OXPHOS, oxidative phosphorylation. ROS, reactive oxygen species. Up and down arrows (↑/↓) indicate an increase and decrease, respectively. ← Indicates the target of regulation. Created in BioRender. Nofal, Z. (2026) https://BioRender.com/f4vvhlk.

p53 partially serves its role by acting within the mitochondria (Figure 3). The p62 protein promotes mitochondrial localization of p53 via its UBA domain [73], and p62 interaction with Parkin prevents its translocation to damaged mitochondria, inhibiting mitophagy [30]. Activation of PKA by p53 enhances inhibitory phosphorylation of DRP1, causing suppression of mitochondrial fission and stimulating their elongation. Suppression of p53 expression reduces mitochondrial length by altering OPA1 and DRP1 expression levels, which improves mitophagy and reduces mt-ROS levels [30]. In mitochondria, p53 raises apoptosis, cuts down mtDNA mutations, and holds mtDNA copy numbers the same. In the case of p53 deficiency, primary mice and human fibroblasts demonstrated a disturbance in mt-ROS homeostasis: decreased superoxide levels in mitochondria and cells and increased hydrogen peroxide levels in cells [74].

mtDNA is contained in the matrix near sites of mt-ROS generation in the mitochondrial membrane, and the absence of histones is believed to make it vulnerable to damage, especially as a result of oxidative damage [75]. P53 acts as a guardian of mtDNA, participating in its repair [76]. p53, through its nuclear transcription factor mtTFA, modulates mitochondrial respiration [74] and is also involved in the assembly and stabilization of the F_1_F_0_-ATP synthase complex [77]. The p53 protein, by inhibiting PDK2, activates PDH, which reduces the conversion of pyruvate to lactate and increases the conversion of pyruvate to acetyl-CoA, which, in turn, promotes OXPHOS [78]. In addition, it activates the transcription of *GLS2*, which encodes mitochondrial glutaminase 2. This enzyme catalyzes the hydrolysis of glutamine to glutamate. This process is essential for synthesizing glutathione (GSH), which reduces cellular ROS levels, and for producing α-ketoglutarate to fuel the TCA cycle [78].

It is believed that the influence of the virus on the sequence of cellular changes as a result of inhibition/activation of p53 allowed it to replicate on the one hand, and to modulate the activation of innate antiviral immune responses on the other [46]. Blood cells from people who died of COVID-19 showed much higher levels of p53. This likely happened because their immune systems were more active compared to patients with milder illness [79].

## 4. Long COVID and Mitochondrial Dysfunction

According to various data, more than 10–36% of patients after the acute phase of COVID-19 developed a wide range of symptoms, from chronic fatigue and brain fog to multiorgan complications. The variety of symptoms from post-COVID problems and the chance they happen after mild COVID-19 cases has created confusion about what they are and how to classify them. The literature employs terms such as “long COVID” and “post-acute sequelae of COVID-19” (PASC) [80,81,82,83,84,85,86,87,88,89,90,91]. Most studies associate the observed symptoms with prolonged mitochondrial dysfunction after COVID-19 infection, along with viral persistence, immune dysregulation, complement dysregulation, prothrombotic inflammation, and changes in the gut microbiome [80,92,93,94,95,96,97,98,99,100,101,102]. SARS-CoV-2 can maintain persistent infection within the host [102,103,104,105], which is considered a driver of long COVID [106]. Persistent SARS-CoV-2 has been detected in patients with long COVID in various tissues for an extended period (up to 2 years) after resolution of acute infection [86,104]. Its persistent reservoirs may include the lungs, brain, liver, heart, olfactory bulb, tonsils, kidneys, and intestinal tissues [86,107]. Persistence of SARS-CoV-2 S, S1, N, and E proteins in tissues and circulation has also been reported [108,109,110] or detection of viral RNA in various tissues of patients with long COVID for a year or more after the acute phase of COVID-19 [110]. The dynamics of changes in the expression of proteins p53, Bcl-2, Bid, Bak and Bax in nasopharyngeal cells in the acute phase of COVID-19 and 6 months later in hospitalized and outpatient patients suggest that after the virus used the cells as a reservoir for its replication, a transition to a chronic disruption of cellular control and suppression of apoptotic processes occurred [111].

Persistence of mitochondrial dysfunction may be supported by immune-mediated damage. The involvement of mitochondria in viral replication likely contributed to the production of autoimmune antimitochondrial antibodies [80,112]. In particular, autoantibodies to epitopes of apoptosis-inducing factor 1 (AIFM1), surfactant locus protein 1 (SURF1) [113], and mitochondrial ribosome-associated GTPase 2 (MTG2) [114] were identified. Notably, patients with long COVID were observed to have higher and increasing titers of antibodies to the N protein compared to the titers of antibodies to the SARS-CoV-2 S protein, which decreased within a year after infection [115]. The immune response to N protein is associated with the development of autoimmune diseases. Among the proteins that are autoantigen targets in humans, predominantly heat shock proteins (including HSP90-β, HSP70, mitochondrial HSP60, and HSPA8), histones (H2B, H3.1–3), and key metabolic enzymes (Glucose-6-phosphate 1-dehydrogenase, Glyceraldehyde-3-phosphate dehydrogenase, pyruvate kinase, and members of the 1st family of aldo-keto reductases) have been identified [116]. Notably, a study of human tissue antigens that could be a potential target for SARS-CoV-2 N protein antibodies using mouse monoclonal antibodies demonstrated that the strongest response was to the mitochondrial pyruvate dehydrogenase complex and transglutaminase 6 (tTG6) [117], which is associated with neurogenesis and motor function [118]. This suggests that oxidative stress and energy imbalance in cells are aggravated by cross-immune reactions. Pyruvate kinase and sorbitol dehydrogenase have been identified as markers that distinguish long COVID with neuropsychiatric disorders from general long COVID [119].

A 9-month study of immune dysregulation in patients hospitalized with COVID-19 showed persistent increases in IL-2, IL-6, IL-10, IL-17A, CXCL9, TNF-α, and IFN-γ compared to healthy controls. However, while IL-2, IL-10, TNF-α, and creatinine levels continued to increase over time, IFN-γ, LDH, CCL2, CXCL9, and CXCL10 levels decreased [120]. Chronic activation of the IFNγ pathway was linked to less T-cell activation and more T-cell exhaustion, hinting at T-cell problems and weaker immune defense [100]. Activation of IL-6 and JAK-STAT signaling pathways was particularly correlated with cardiorespiratory or multisystem symptoms in long COVID and was observed over a prolonged period, with currently available data extending up to 2 years [97,100].

Although the RAS imbalance in COVID-19 patients was initially thought to be short-term, further research has shown that it may be more long-term [26]. Dysregulation of *MAS1*, *ACE2*, and *ACE* expression in peripheral blood mononuclear cells (PBMCs) was found to persist for 300 days after the acute phase of the disease. Lower *ACE2* expression was seen in COVID-19 patients who needed oxygen in the hospital and in those with type 2 diabetes. In contrast, higher *ACE2* expression was found in patients without other health issues and in those with high blood pressure when compared to the control group. In all patient categories, *MAS1* expression in PBMCs was higher during the acute phase than after COVID-19 [121]. In addition, cases of anti-idiotypic antibodies to ACE2 have been reported in patients who recovered from COVID-19 and in those vaccinated against SARS-CoV-2, leading to immune damage to ACE2-expressing cells [122,123,124].

The clinical manifestations of long COVID are influenced by multiple factors [125], including gender and the severity of COVID-19. Women were significantly more likely than men to develop post-COVID complications [126], the symptoms of which, although diminishing, persisted for up to 36 months, with various gender-specific predictors determining symptom progression [127]. Neuropsychiatric complications, including cognitive dysfunction, depression, and anxiety, are the most common post-COVID complications, accounting for 30 to 50% of individuals with long COVID [128]. Individuals with long COVID also had reduced levels of circulating cell-free mtDNA (ccf-mtDNA), reflecting impaired mitochondrial homeostasis [96,101]. Since higher ccf-mtDNA levels were associated with better cognitive performance, their decrease was associated with potential deterioration of cognitive function [101]. Moreover, women predominated in the group of individuals with neuropsychiatric complications [129], which is associated with gender-specific differences in the immune system response, in particular leading to the development of chronic fatigue [129,130]. Myalgic encephalomyelitis/chronic fatigue syndrome (ME/CFS) and long COVID with neuropsychiatric symptoms share similar symptomatology and common pathophysiological mechanisms, which are based on immune-mediated injury and neuroinflammation along with mitochondrial dysfunction and a proinflammatory gut microbiome [129]. A research study looked at people with serious COVID-19 and lasting lung function issues. It found special immune and inflammatory patterns in those with severe cases and long COVID. Fatigue, neurocognitive symptoms, and exercise intolerance were most common in patients with long COVID, mostly women, who had decreased total white blood cell counts, lactate dehydrogenase, and IL-6 levels, while LAMP3 (lysosomal-associated membrane glycoprotein 3), CKAP4 (cytoskeleton-associated protein 4), and KRT19 (keratin 19) were overexpressed compared with patients who had severe COVID [131]. Studies have shown that chronic fatigue persisted for two to four years after the disease [132,133,134]. However, in general, the severity of acute COVID-19 infection is a key factor in the manifestation of long-term adverse health effects, although the risk decreases significantly within 3 years of the disease [135].

A number of studies also suggest a genetic predisposition to the development of long COVID [136,137]. Although limited, one study demonstrated a genetic predisposition to mitochondrial dysfunction in patients with severe long COVID [137]. In another study, genes associated with the immune response to infection were frequently found in patients with severe long COVID, while in the group with fatigue as the primary symptom, genes associated with the MAPK and JNK signaling cascades, as well as metabolic processes involved in mitochondrial function and cellular respiration, were predominant [136].

Studies of mitochondrial gene expression in autopsy tissues of COVID-19 patients have shown downregulation of mitochondrial OXPHOS genes in the lung, heart, kidneys, liver, and lymph nodes not only in the acute phase of the disease but also long after virus clearance [29,138,139]. Mitochondrial dysfunction in various organs and tissues [29,138,139], likely also contributed to the development of some of the phenotypic manifestations of long COVID (Table 2).

The connection between lasting changes in mitochondria and certain long COVID types is eagerly awaited, but proving a clear cause is difficult. This is due to the wide range of long COVID symptoms, from fatigue to neurological issues, and the lack of specific tissue data. Mitochondrial issues likely act as a hidden cause that leads to different long COVID symptoms in patients, based on their own genes, metabolism, or tissue specifics.

SARS-CoV-2 caused an increase in miR-2392, which can enter mitochondria and stop mitochondrial RNA polymerase, leading to a decrease in mtDNA gene transcription. Furthermore, miR-2392 suppresses the mRNA translation of nuclear mitochondrial genes. Its main targets were mainly transcripts of mitochondrial metabolism genes, OXPHOS, mitochondrial biogenesis genes, mitochondrial fusion, and mitophagy genes [29]. Expression of the CEBPB transcription factor mRNA can be regulated by factors such as IFN-γ, IL-6, EGF, and bFGF, as demonstrated in Hep3B cells. IFN-γ had the strongest effect on initiating *CEBPB* mRNA expression [145]. *CEBPB* gene expression was negatively correlated with the expression of OXPHOS genes encoded by both mtDNA and nuclear DNA (nDNA) in most human tissues [146]. These studies suggest that persistent inhibition of OXPHOS transcripts is associated with progressive changes in pathways associated with decreased muscle function and fatigue in the post-COVID period [138]. This is also confirmed in patients with 3-month long COVID, who showed a sharp decrease in skeletal muscle mitochondrial succinate dehydrogenase (SDH) activity and increased glycolysis after induction of post-exertional malaise [147]. A decrease in SDHB activity can lead to succinate accumulation and inhibition of the enzymatic activity of HIF-1α prolyl hydroxylases (PHDs), thus stabilizing HIF-1α, creating a pseudo-hypoxic state [148]. Increased *HIF1A* mRNA expression in monocytes correlated with the severity of COVID-19 [149] and persisted for up to 4 months in patients with moderate COVID-19 [138]. Furthermore, using the infected human bronchial epithelial cell line BEAS-2B, it was demonstrated that the Wuhan SARS-CoV-2 strain and the S protein activate HIF-1 in cells, likely by blocking the prolyl hydroxylation of HIF-1 through the conversion of cellular 2-oxoglutarate to 2-hydroxyglutarate. HIF-1 activation triggered the production of TGF-β, which induces the expression of immune checkpoint proteins such as galectin-9, programmed death ligand 1 (PD-L1), and indoleamine 2,3-dioxygenase, an enzyme involved in the production of the immunosuppressive amino acid L-kynurenine. These immune checkpoint pathways are capable of suppressing both helper and cytotoxic T cell activity [150]. A positive correlation of IL-6 and JAK-STAT signaling pathways with PD-1 signaling, demonstrated in blood samples over 180 days, confirmed chronic inflammation in patients with long COVID [100]. Analysis of PBMC samples from patients with mild and severe forms of the disease collected at 2–4 and 4–12 months post-infection also demonstrated a decrease in the levels of OXPHOS transcripts encoded by mtDNA in all cases, as well as by nDNA in severe cases [138]. Another study of mitochondrial dysfunction in PBMCs and granulocytes from long COVID symptomatic patients at 8 months or more demonstrated that 8 of 65 differentially expressed genes in PBMCs were mitochondria-related: *MT-CYB*, *MT-CO3*, *MT-TV*, *MTND2P28*, *MTND6P3*, *MTCO1P40*, *MTCO2P22*, and *MTCO2P12* [151].

Strong induction of OXPHOS genes in human lung samples observed during the acute phase, along with immune gene expression and mitochondrial stress response, remained elevated, indicating ongoing inflammatory signaling [29,138]. Meanwhile, RAAS and aging-related pathways were activated in the lungs, heart, and kidneys, and PANOptosis was strongly induced in the lungs and heart [138]. A proteomic study of blood from patients with neurologic symptoms of long COVID 3–9 months after recovery showed that mitochondria play a role in the symptoms. They exhibited altered levels of mitochondrial ETC proteins and elevated serum COX7A1 concentrations, reflecting mitochondrial damage, which positively correlated with post-COVID symptoms [93]. Altered expression of the mitochondrial genes *COX7C*, *COX7A2*, *ATP5PO*, *ATP5F1C*, and *NDUFB8* also persisted in the lungs and brain of recovered patients, ranging from excessively high levels at 12 weeks post-SARS-CoV-2 infection to gradually declining by week 24. The *EGFR* gene plays a crucial role in the later stages of COVID-19. Its expression stayed down for at least 12 weeks after recovery. Its activation by the SARS-CoV-2 S protein triggers phosphorylation of canonical extracellular signal-regulated kinase 1/2 (ERK1/2) and the AKT signaling pathway in lung tissue and contributes to the inflammatory cascade observed in some COVID-19 patients [152].

Research on mitochondrial issues in patients who got better from COVID-19 over a period of 4 to 13 months showed that these issues differed with the severity of the disease. Patients with severe COVID-19 demonstrated a persistent imbalance between mitochondrial fusion proteins (MFN2 and OPA1 were downregulated) and fission proteins (DRP1 and FIS1 were upregulated) in the mitochondria, along with impaired structural integrity of the ETC, higher levels of ROS, persistent serum levels of IL-1β, decreased levels of IFN-α2 and IL-27, and a trend toward increased circulating levels of 3-hydroxybutyrate compared with patients with mild COVID-19 [153]. The concentrations of mitochondrial regulatory proteins, including PINK1, dynamin-1-like protein (DNM1L), and MFN2, as well as chemokine ligand 18 (CCL18), IL-6, and TNF-α, were significantly higher in the serum of recovered patients with pulmonary COVID-19 after 3 months compared with the control group, while the concentration of IFN-α was reduced [154]. DNM1L levels could be used to assess the severity of COVID-19, as they were significantly higher in deceased patients than in those who recovered from severe disease, while TOMM20 and TOMM22 showed the opposite trend, generally reflecting mitochondrial dysfunction [155]. Mitochondria from patients with long COVID show signs of severe mitochondrial stress: disrupted structure (significant swelling, cristae disruption, and overall irregular morphology), oxidative stress markers (elevated levels of superoxide dismutase 1, F2-isoprostanes, and malondialdehyde, decreased levels of the antioxidant coenzyme Q10), impaired mitophagy (elevated levels of autophagic cysteine peptidase 4B), and reduced levels of circulating extracellular mtDNA [80,95,96].

Gene expression data from blood samples of patients with COVID-19 showed that *TP53* expression levels dropped after severe SARS-CoV-2 infection. This decline lasted for up to 24 weeks with no signs of improvement. In milder cases, the decline in *TP53* expression was smaller and gradually recovered. However, of the six disrupted p53 signaling pathways, those associated with apoptosis, signaling, and DNA damage response remained impaired even after 24 weeks [2]. However, it has also been reported that circulating extracellular vesicles in patients with long COVID—which induce aberrant expression of the transcription factor RUNX2 and activate the stress proteins Sestrin 1 and 2 (SESN1/SESN2)—lead to persistent hyperactivation of the p53/p21 signaling pathway in lung cells, endothelial cells, and mesenchymal stem cells. This results in impaired cellular metabolic adaptability, specifically a reduction in mitochondrial respiration and the inhibition of ATP production [156].

Mitochondria in leukocytes of patients with post-COVID-19 sequelae had reduced ΔΨm levels even after 11 months compared to healthy controls. Moreover, men had a better ΔΨm recovery after 11 months than women, which may be related to the influence of estrogen [157]. ΔΨm was lower in the mitochondria of various immune cells in patients with long COVID showing different symptoms: in CD56 bright natural killer cells, mainly in those who felt dizzy, and in CD4+ lymphocytes in patients with worsening breath issues. IFN-γ production in response to cell stimulation with lipopolysaccharide was significantly lower in these patients [99]. CD14+/CD16+ monocytes from patients with long COVID suffering cardiovascular complications revealed lasting energy and mtDNA damage over a one-year period [158]. Furthermore, PBMCs from patients with post-COVID-19 sequelae showed altered activity of Complex V, which also contributes to the generation of ΔΨm. Complex V functions in a unique way rather than the typical ATP production that relies on the proton gradient. It uses ATP to move protons back into the intermembrane space to support the decreasing ΔΨm [159]. Analysis of gene expression differences in the PBMCs of patients one year after COVID-19 showed a clear molecular pattern, especially in those who had severe COVID-19. The central genes of this signature were *H3-5/H3F3C*, *H3C13/HIST2H3D*, *JUN*, *EGR1*, *NOTCH1*, and *SQSTM1/P62*, with the autophagy-related gene *SQSTM1/P62* identified as a distinctive central gene interacting with key genes associated with inflammation, apoptosis, and cancer progression [160]. Elevated expression of SQSTM1/p62 suggests that the p62 protein enhances the localization of p53 to mitochondria. Consequently, the accumulation of p53 in mitochondria suppresses mtDNA transcription and blocks the mitochondrial unfolded protein response. This leads to a decrease in ΔΨm, a drop in ATP levels, and a sharp increase in ROS, which triggers the mitochondrial apoptosis pathway.

Impaired mitophagy was one of the consequences of SARS-CoV-2 cell damage [161]. Impaired mitophagy is also a cause of pulmonary fibrosis in various diseases, leading to the accumulation of damaged mitochondria, increased mt-ROS, ferroptosis, and cellular senescence [141,162,163]. Functional lung injury and fibrotic changes after COVID-19 were observed in almost 55% of patients with long COVID after 12 weeks [164]. One year after the disease, they persisted, according to some data, in half of those with severe COVID-19, and in 22.7% and 27.3% of patients with mild and moderate forms of the disease, respectively [165], while another study found residual inflammatory and profibrotic changes on computed tomography in almost 70% of patients who had severe and critical pneumonia, although it noted that their level had decreased [166]. After 3 years, persistent lung changes associated with respiratory symptoms and decreased diffusion capacity still persisted in more than a third of patients [167]. Mouse models have shown that thymidine phosphorylase may play a leading role in lung injury, fibrosis, and tumorigenesis as a result of ACE2 inactivation by the S protein through activation of the STAT3 signaling pathway and remodeling of the inflammatory microenvironment [168]. STAT3 activation may likely lead to PINK1-Parkin-mediated mitophagy, along with increased TGF-β1 and EMT levels in bronchial epithelial cells, similar to what occurs with cigarette smoke [169]. PINK1-Parkin-mediated mitophagy requires p62 interaction with the microtubule-associated protein light chain (LC3), which was disrupted by the viral NSP5 and ORF3 proteins [170,171]. Serum mTOR levels exceeding the threshold of 6.15 ng/mL were also associated with the presence of fibrotic changes in the lungs after COVID-19 at 12 weeks [172].

Functional and structural changes in mitochondria caused by SARS-CoV-2 exposure contributed to accelerated aging [9,29,60].

Elevated basal NF-κB levels correlated with proinflammatory features of aging and age-related diseases. Furthermore, since Ang II accumulation can also increase NF-κB levels, SARS-CoV-2 suppression of ACE2 activity leads to additional NF-κB activation [46]. It is important to know that checking the RAAS imbalance in long COVID patients by looking at Ang II levels in the blood is not possible. This imbalance happens in the tissues. So, the study by Lange-Tal T. et al. [173] cannot prove that there is no long-lasting RAS imbalance. The IFN-γ signaling pathway and the canonical NF-κB signaling pathway (particularly TNF-α-mediated) were among the most enriched signaling pathways in individuals with long COVID, and IFN-γ was identified as a promising biomarker for long COVID [174]. The level of cytokine response in long COVID was also associated with disease severity. Levels of cytokines such as IL-17, IL-10, IL-4, IL-2, TNF-α and IFN-γ increased after mild/moderate disease, whereas after severe COVID-19, only levels of IL-17, IL-2, and TNF-α increased [175]. Notably, elevated levels of TNF-α, IL-1β, and IL-6 were observed after 10 months [176], although a decrease in IL-6 levels was reported 30 days after recovery [175]. CEBPB may regulate *ACE2* gene transcription; bioinformatics analysis demonstrated both positive and negative correlations of CEBPB with *ACE2* gene transcription in several cancer types [177]. These data are also of interest given that IFN-γ and IL-6 remain upregulated in long COVID, promoting chronic inflammation. Furthermore, IL-6 promotes immune evasion by inducing PD-L1 stabilization [28].

A 3-year post-COVID-19 study of DNA methylation-based aging revealed accelerated aging in women, but not in men [178]. DNA methylation, as one of the mechanisms of epigenetic regulation of gene expression [179], also contributed to the development and maintenance of long COVID. In circulating leukocytes of individuals who recovered from COVID-19, DNA methylation changes were partially maintained for at least 1 year [180]. Alterations in transcriptional and epigenetic programs in monocytes (and their precursors) were detected 4–12 months after recovery from COVID-19 and were annotated to immune response and inflammation genes, in particular *IL21R*, *MAPKAPK2*, *TNIP2*, *CREB1*, and *MMP1*, as well as pathways associated with monocyte activation and cytokine production [181]. Moreover, SARS-CoV-2 infection did not change the average DNA methylation level across the genome in blood samples, but affected only the loci of genes associated with COVID-19, mainly those related to the immune response: cytokine/chemokine and receptor genes (including *IL-10*, *IL-1β*, *CXCR2/5/6*), interferon-stimulated genes (*IFIT3*, *ISG2*, *IFI27*, and *OAS2*), signaling genes (*TRAF2*, *ZAP70*), *IRF7*, *BCL6*, *MX1*, and *TNF* [182]. DNA hypermethylation in the promoter region of a gene leads to suppression of its expression, while hypomethylation increases gene expression [179]. During severe COVID-19, there was an increase in the methylation of interferon genes and a decrease in inflammatory genes, as well as an increase in DNA methylation age [183]. Changes in mtDNA gene methylation following SARS-CoV-2 infection disrupted the assembly of the NADH dehydrogenase complex, CI and CIV of the ETC, OXPHOS, the TCA cycle, ketone biosynthesis, and ATP metabolism [155]. In the acute phase of the disease, DNA methylation levels positively correlated with its severity [155,180]. However, asymptomatic and mild COVID-19 patients also showed persistent changes in DNA methylation patterns 7 months after recovery. In particular, hypermethylated cg17126990 (*AFAP1L2*) and cg25483596 (*PC*) showed the strongest positive association with disease status. The *AFAP1L2* gene encodes a protein, actin filament-associated protein 1-like 2, that can bind to SH3 domains, activate protein tyrosine kinase, and help improve the EGFR signaling pathway. The transcription factors ELAVL1 and PABPC1 bind to a region containing cg17126990. ELAVL1 was upregulated in COVID-19 patients and is also known to participate in inflammation by regulating mRNA stability, splicing, and translation. The cg25483596 site is annotated with the *PC* gene, encoding pyruvate carboxylase, a key mitochondrial enzyme involved in gluconeogenesis, lipogenesis, insulin secretion, and glutamate synthesis [184]. Notably, the methylation patterns of COVID-19 patients with varying disease severity were inconsistent across studies [184,185]. The difference in immune response between men and women to COVID-19 could be associated with different DNA methylation patterns, particularly in the X-linked genes *ACE2*, *CA5B*, and *HS6ST2*. Cg21598868 in the *ACE2* gene showed different levels of methylation based on COVID-19 status in both men and women [186].

## 5. Discussion

Although the COVID-19 pandemic ended four years ago, it has left its mark in the development of post-COVID complications, known as long COVID. Even the lowest reported estimate of 10% of COVID-19 survivors suggests that more than 70 million people worldwide suffer to varying degrees from reduced quality of life, loss of productivity, and are at risk of developing various diseases, primarily neurodegenerative ones [187]. An initial analysis of the clinical manifestations of long COVID identified nearly 200 symptoms affecting the functioning of more than 10 body systems [188]. Molecular signatures and phenotypic manifestations of COVID-19 may vary significantly depending on the severity of COVID-19 and the presence of comorbidities [85,189]. Meta-analysis data suggest that symptoms such as neurological, psychological, cardiovascular, muscle, and general weakness show an increasing trend compared to dermatological, respiratory, and gastrointestinal symptoms [87].

Mitochondria have emerged as a central component of the pathology of not only COVID-19 but also long COVID. The virus not only exerted direct effects through its proteins, exploiting mitochondria for its replication through altered cellular metabolism and suppression of the innate immune response, but also likely exerted indirect effects through suppression of the ACE2/Ang (1–7)/MasR axis, targeting mitochondria as components of the RAAS, as well as through suppression of the p53 protein, which is also involved in mitochondrial regulation. A comprehensive multi-omics analysis of transcriptomic, proteomic, and metabolomic data from various tissues of Syrian hamsters and human cohorts [138] clearly demonstrated the persistence of altered RAAS and activation of aging-related pathways in long COVID in the heart, lungs, and kidneys. Suppression of RAAS, along with suppression of OXPHOS, HIF-1α, immune system, and neuronal metabolic pathways in autopsy brain samples from deceased COVID-19 patients, suggests that both mitochondria and RAAS were affected in the brain of patients with severe disease [138]. In patients with milder COVID-19, this lesion could contribute to increased oxidative stress along with impaired mitophagy. Altered p53 expression was observed not only during SARS-CoV-2 infection [2,79,111] but also long-term [111]. The nature of the response was determined not only by the severity of past COVID-19 [111] but also by the modes of expression regulation and the stress context in different cell types [156], which determine mitochondrial dysfunction. Furthermore, the persistence of viral proteins could potentially support mitochondrial fragmentation, increased Ca^2+^ levels, and a decrease in ΔΨm in cells from patients with long COVID, as was observed with the effect on human primary ventricular cardiomyocytes [143].

Yet, the question remains as to why cognitive impairment and emotional distress were the leading symptoms in the post-COVID period. It is more reasonable to think about the effects of lung inflammation and the cytokine storm that caused the disease to be severe. Post-COVID syndrome happened not just in patients with severe COVID-19. There were also cases of long COVID in people who had mild or no symptoms at all [190]. What specifically determines the primary pathogenesis of COVID-19, leading to the development of neurodegenerative processes? What characteristics of nervous tissue make it more sensitive to the long-term effects of the virus?

As noted above, the brain can serve as a refuge for persistent SARS-CoV-2, where it can remain hidden for extended periods [86], similar to other viruses such as Ebola, herpes, and HIV, which exploit the brain’s immunological privilege [191,192]. A key trait of nerve cells is that they cannot divide after they mature, so if neurons are exposed to a virus, they undergo apoptosis and die. Suppression of mACE2, a protective factor against endoplasmic reticulum stress, by the SARS-CoV-2 S protein, along with increased viral protein synthesis and impaired autophagy, may stimulate endoplasmic reticulum stress, leading to impaired proteostasis [193,194], which leads to mitochondrial dysfunction in neurons and may also trigger neurodegenerative processes [30]. In addition, the viral S protein fragment has toxic amyloidogenic properties and the ability to co-aggregate with beta-amyloid, potentially mimicking the early stage of Alzheimer’s disease (AD), with further progression of amyloidogenesis, tauopathy, neuroinflammation and dysregulation of immune responses, which is believed to aggravate the pathology of AD and provoke long COVID [195]. In turn, beta-amyloid can penetrate into mitochondria and, through the suppression of oxidative phosphorylation, stimulate neuronal apoptosis [196]. Impairment of mitochondrial biogenesis and mitophagy provoked by SARS-CoV-2 and its S protein is a key point uniting COVID-19, aging and neurodegenerative diseases [161,197].

Long COVID was characterized by astrogliosis and impaired astrocyte function, which increased in size and changed morphology [198,199,200]. Impaired astrocyte function resulted in glutamate accumulation in the synaptic cleft, causing excitotoxicity associated with the uncontrolled influx of Ca^2+^ through N-methyl-D-aspartate (NMDA) receptors into neurons and mitochondria, inducing the release of apoptosis activators into the cytosol, leading to neuronal death [201,202,203]. Indeed, in individuals with long COVID with persistent cognitive symptoms and increased fatigue, cortical thinning was found, occurring both due to a decrease in gray matter (neurons) and white matter caused by damage to oligodendrocytes and demyelination of axons [204,205].

The virus has shown that it can enter the brain and affect neurons, astrocytes, and oligodendrocytes [206,207]. The virus does not spread much in the brain, and its harmful effects might affect only a few infected cells. This may not lead to clear changes in behavior. Most problems come from indirect causes like RAAS, inflammation, and issues with oxidative phosphorylation. In most cases, the manifestations of long COVID remain non-specific, making it difficult to assess the overall reduction in cell survival caused by the virus. COVID-19 models have shown that the virus can enter glutamatergic neurons, causing them to die [187], but there are very few of these cells. This suggests that the brain problems are related to function and not to any physical damage, so they might be treated with drugs.

We earlier explained how the p53 expression changes play a part in COVID-19 infection, mainly in mitochondria. Normally, p53, by inhibiting PDK2, activates PDH, which reduces the conversion of pyruvate to lactate and increases the conversion of pyruvate to acetyl-CoA, which, in turn, promotes OXPHOS [78]. In addition, it activates the transcription of mitochondrial glutaminase 2 (GLS2), which catalyzes the hydrolysis of glutamine to glutamate, which is necessary for the synthesis of glutathione (GSH), which reduces ROS levels in cells, and the production of α-ketoglutarate, which enters the TCA cycle [78]. When the p53 level in the cell lowers, ROS is produced, ATP production in the TCA cycle is reduced, and making glutamate from glutamine gets slower. This may result in disruption of the brain’s main excitatory glutamatergic system, which plays a central role in cognitive function. Long-term changes in cellular function may also be caused by DNA methylation. Hypermethylation of the *AFAP1L2* and *PC* genes was linked to a patient’s worsening condition with COVID-19. In the case of the *PC* gene, which encodes pyruvate carboxylase, a key mitochondrial enzyme, in addition to gluconeogenesis, lipogenesis, and insulin secretion, glutamate synthesis was also reduced [184]. This further suggests the possible involvement of disturbances in the glutamatergic system and associated learning and memory impairments in long-COVID.

Long-term changes are seen in RAAS activity. There is an imbalance between its two parts. Ang II levels go up, while mACE2 levels go down. These changes cause problems with mitochondria and also affect important neurotransmitter systems. This can lead to depression and brain diseases, like Alzheimer’s disease. ACE2, through its regulation of glutamate and Gamma-Aminobutyric Acid (GABA) transporters, plays a key role in maintaining the balance between the GABA and glutamate systems. Furthermore, ACE2 activity is also associated with the dopaminergic system of the brain [23]. Dysregulation of the RAAS leads to deterioration of dopaminergic neurons in the nigrostriatal system by causing AT1R activation. This activation triggers the cellular NADPH oxidase-superoxide pathway and increases Ca^2+^ release, provoking oxidative stress and neuroinflammation [208]. SARS-CoV-2-induced downregulation of ACE2 levels also impaired the function of transporters that include ACE2, which reduced the synthesis of dopamine and serotonin (5-HT), also affecting the functioning of the serotonergic system, which is involved in emotional responses, memory, and neurogenesis [23,209]. ACE2 is important for the acetylcholinergic system. This system helps with many behaviors like learning, memory, attention, being alert, and automatic muscle movements. In the brain, acetylcholine works as a chemical messenger and influences two main kinds of receptors: nicotinic nAChRs and muscarinic mAChRs. Importantly, stimulation of nAChRs can increase the release of neurotransmitters such as glutamate, GABA, and dopamine, participating in the synchronization of neuronal activity [210]. Nicotine has been shown to activate ACE2 in neurons, glial cells, and endothelial cells through nAChR activation. According to the “nicotine hypothesis” of Changeux et al. (2020) [211], the SARS-CoV-2 virus can directly interact with nAChR and block its binding to acetylcholine. This will not only disrupt the harmony of neurotransmitter interactions in the body, but also cause the development of a variety of neurological symptoms associated with long COVID, including systemic mitochondrial dysfunction, as several nAChR subtypes have been identified on the outer mitochondrial membrane [212]. These mitochondrial nAChRs activate intrinsic mitochondrial kinases such as Ca-calmodulin-activated kinase II, Src kinases, and phosphatidylinositol 3-kinase, which collectively regulate mitochondrial signaling and metabolic pathways, providing protection against pro-apoptotic factors such as Ca^2+^ and ROS, and sustaining ATP production for a long time [213]. Blockade of these receptors by the virus will cause an increase in ROS and oxidative stress. The fact that disturbances in the brain’s acetylcholinergic system are present in patients with long COVID has also recently been confirmed [214].

Interestingly, among brain structures, particularly high mtDNA copy numbers are found in neurons of the frontal and cingulate cortex [32], brain structures that play an important role in learning and memory. It is possible that prolonged post-COVID suppression of OXPHOS transcripts encoded by mtDNA and nDNA in the brain of individuals who have recovered from severe COVID-19 and the reduced levels of cc-mtDNA [101,138], characteristic of the post-COVID state, sharply impairs the functioning of these brain structures.

Recently, many excellent reviews have appeared devoted to analyzing the possible causes of long COVID. Many authors feel that long COVID is not caused by a virus directly affecting cells, but is more closely related to long-term neuroinflammation, nerve damage, mitochondrial issues, calcium buffer problems, lowered energy metabolism, and other similar issues. We believe long COVID is caused by issues in the systems that are found in organisms, cells, and their parts, which control important functions for survival during changes in the environment. At least two systems—the RAAS and the acetylcholinergic system—can play this role. They closely interact with each other and with mitochondria, which are present in all cells of the body except erythrocytes. In U373 cell lysates, a possible interaction of ACE2 with α7nAChR was detected, and α7AChR activation effectively inhibited the interaction of SARS-CoV-2 with ACE2 [215]. It is also worth considering that in the lungs, kidneys, circulatory system, and brain, AChRs are not only very widely distributed but also often localized in the same cells that express ACE2 [211]. In light of the above, it seems advisable to base the long-COVID treatment strategy primarily on adjusting the activity of these two systems. ACE2 activators such as DIZE, which is not approved as a human drug [216], or the developmental H4 [217] could be used. Cholinergic receptor agonists [214], and perhaps even a nicotine patch, also show promise. To lessen serious brain symptoms and help memory in long COVID, we need to balance the glutamate and GABA systems. This will stop cell damage and adjust NMDA and α-amino-3-hydroxy-5-methyl-4-isoxazolepropionic acid (AMPA) receptors. Taking metformin can help with this to some degree. The stress proteins HSP70 and YB1 have a systemic, normalizing, nonspecific effect; however, they have not yet been registered as therapeutic agents [218]. However, these efforts might not be enough. The mitochondria and how they create mtDNA are influenced. This points to mitochondrial dysfunction in long COVID. Altered astrocyte function in long COVID disrupts their interaction with neurons, which normally receive healthy mitochondria from astrocytes to replace dysfunctional ones. It is conceivable that neurons deprived of their usual support from astrocytes may switch to a different mode of activity. Mitochondrial dysfunction, along with genomic instability, telomere shortening, epigenetic reprogramming, loss of proteostasis, impaired regulation of nutrient uptake, and altered intercellular communication, can also trigger cellular senescence [30,219], which is often found in long COVID. Mitochondria in senescent cells not only exhibit reduced ΔΨm, impaired ETC function, increased mt-ROS production, and impaired mitophagy, but also have altered structure and shape [30]. In turn, exceeding the threshold level of senescent cell load leads to accelerated aging and disease development [219]. The combined data presented inevitably leads to the conclusion that in long COVID, it is the mitochondria that require therapeutic intervention.

Currently, there are no specific therapeutic agents directly targeting the treatment of long COVID. This is due to the variety of its symptoms and the challenge of developing and conducting drug tests after the COVID-19 pandemic ended. Consequently, the treatment applied to long COVID patients is extrapolated from therapies for other diseases characterized by mitochondrial dysfunction. It focuses primarily on restoring bioenergetics and reducing systemic inflammation. Interventions can target the main issues of long COVID, such as problems with iron, immune system troubles, and harmful substances in the blood that can affect cell function and immunity. Agents called mitoprotectors help support mitochondria. They are in the final phase of testing. These agents include coenzyme Q10, L-carnitine, NAD boosters (like NMN and nicotinamide riboside), and antioxidants. There are also special rehab plans that often use a mix of these treatments [80]. Mitochondrial oxidative stress can also be reduced using mitochondrion-targeted catalytic antioxidants such as MnTBAP, Mito-TEMPO and mitoquinol. Effective treatment can also be obtained with mitochondrial Permeability Transition Pore (mPTP) inhibitors such as NIM811 [92]. The detailed treatment approaches for long COVID are covered in a number of articles and recent reviews [80,220,221,222,223,224,225]. It should be noted that this does not preclude the treatment of comorbid chronic diseases affecting patients with long COVID. A number of authors argue in favor of new interventions that address brain symptoms related to long COVID. They suggest using antioxidants like Tempol, taking vitamins A, C, D, and E, and trying neurogenic stem cell and mitochondrial therapy [226]. Adding foods with polyphenols and medium-chain triglycerides to the diet is suggested. These provide another energy source by avoiding broken parts of the mitochondrial respiratory chain. Mitochondrial therapy is noteworthy. It is already used for different human illnesses and has proven it can improve outcomes in ischemia–reperfusion injury cases [227]. Minovia Therapeutics is in the final stage of clinical trials for this development. This project helps cells work better by giving them healthy mitochondria from donor placentas. This method seeks to tackle problems with mitochondrial function, particularly in aging, which is faster in long COVID [223], It hints that mitochondrial therapy could be helpful for long COVID as well. Experimental studies on animals and nerve tissue cultures have demonstrated the ability of exogenous mitochondria to penetrate neurons, astrocytes, and microglia [228,229]. In this context, the feasibility of healthy mitochondria entering the brain following intranasal administration was investigated [229,230]; crucially, the efficacy of this procedure was demonstrated in models of Parkinson’s disease [231], Alzheimer’s disease [230,232], post-stroke cognitive impairment [233], ischemic brain injury [234], depressive- and anxiety-like behaviors [235] and other pathologies associated with memory loss, neurological deficits, and mitochondrial dysfunction [235]. Given that the pattern of brain structural damage in acute COVID-19 proved remarkably similar to that of Alzheimer’s disease, particularly regarding the localization of beta-amyloid plaque deposits [236], which, as previously noted, are also found in a subset of patients with long COVID, it is reasonable to hypothesize that intranasal transplantation of healthy mitochondria would yield positive results in clinical trials involving long-COVID patients [226]. Many unresolved issues exist for most new long-COVID treatments. These issues include effectiveness, duration of effects, safety, delivery methods, standard methods, and side effects. Despite the challenges of treating post-COVID issues, we believe that effective treatments for long-COVID patients will be available in the near future.

## 6. Limitations

This review has limitations mainly because data on post-COVID effects keep being published, and the studies included vary in length—from a few months after infection to several years.

Furthermore, existing publications differ both in the sizes of the patient samples studied and in the parameters examined. At the same time, a number of symptoms (such as increased fatigue, head noise, and impaired concentration) are difficult to quantify.

Autopsy samples from those who died of severe COVID-19 show serious problems with mitochondria in cells from many organs. However, post-COVID syndrome can also happen in people who had a mild case of the disease. So, we cannot say there is one way that mitochondria are affected in all these patients. Checking mitochondrial problems in blood cells tells us about ongoing body issues, but it does not let us see damage in the tissues of certain organs directly [80,159,237].

During the pandemic, different SARS-CoV-2 strains were present. The vaccines used were also varied and vaccinations did not start right away. So, many researchers have not taken these factors into account. Assessing their precise contribution based on available data is extremely difficult; consequently, they were not addressed in this review. The development of certain complications, particularly those linked to neurodegenerative processes, unfolds over a prolonged period. As a result, an increase in other risk factors contributing to such pathologies may mask the role of a prior SARS-CoV-2 infection in their onset. This makes it hard to connect timing and causality between COVID-19 and these diseases, even though more studies are finding these links. This review discusses the existence of this link based on available clinical data and observations. Large-scale studies are needed to establish precise causal relationships regarding mitochondrial dysfunction across the various phenotypic manifestations of long COVID. Such research would help improve therapeutic strategies and facilitate the development of effective treatment protocols for patients with post-COVID syndrome.

## Figures and Tables

**Table 1:** Function of SARS-CoV-2 proteins and their impact on mitochondria.

SARS-CoV-2 Proteins	Function and Effect on Mitochondria	References
**Non-structural proteins**		
NSP1	Interacts with DNA polymerase α protein. Suppressor of host IFN signaling [9,48]	
NSP2	May enhance miRNA-mediated suppression of cellular transcripts. Inhibitor of prohibitin (PHB), a chaperone for ETC proteins, interacts with PHB1 and PHB2 isoforms. It suppresses IFN production by binding to the GIGYF2 protein, a repressor of *Ifnb1* transcript translation. NSP2 influences VDAC2 biogenesis and increases the amount of stomatin-like protein 2 (STOML2), [8,9] which contributes to a decrease in apoptosis. Together with NSP4, it interacts with Hsp70, components of the inositol triphosphate receptor (IP3R) ubiquitination system, and proteins necessary for vacuolar ATPase biogenesis, affecting mitochondrial Ca^2+^ homeostasis.	
NSP3	NSP3 has protease activity; together with NSP4 and NSP6, it causes ER membrane curvature; together with NSP4, it participates in the formation of the DMV; it is localized in the ER and mitochondria; and it interacts with the N protein during SARS-CoV-2 gRNA replication and virion assembly. NSP3 cleaves host ubiquitin and the ubiquitin-like protein ISG15, affecting innate immunity.	[9]
NSP4	NSP4 is involved in the formation of DMV; together with NSP2, through the recruitment of ERLIN1/2 and RNF170 proteins, it influences the homeostasis of mitochondrial Ca^2+^ at the sites of contact between the ER and mitochondria; it interacts with proteins of the Translocase of the Inner Membrane (TIM) complex of mitochondria and disrupts import.	[9,48]
NSP5	NSP5 has protease activity; interacts with histone deacetylase 2 (HDAC2); participates in the suppression of the host’s innate immune response and IFN synthesis by disrupting the RIG-I-MAVS complex; activates the NF-κB signaling pathway through SUMOylation of the MAVS signalosome, which increases its stability and further triggers the NF-κB pathway; cleaves melanoma-associated antigen D2 (MAGED2), which is involved in cell cycle regulation and stress response pathways (including hypoxia). Together with the N protein, it blocks the formation of “stress granules”.	[9,48]
NSP6	NSP6 is involved in the biogenesis of DMV; its oligomerization and formation of the amphipathic helix zips ER membranes and forms connectors as a result of the activity of NSP3 and NSP4.	[9]
NSP7	RNA polymerase cofactor. NSP7 interacts with the Complex (C) I assembly chaperone NDUFAF2 (NADH dehydrogenase [ubiquinone] 1 alpha subcomplex assembly factor 2) and CYB5B (Cytochrome b5 type B); with components of the GPCR signaling pathway, such as guanine nucleotide binding protein beta-1 subunit (GNB1), rat sarcoma virus homolog A family member (RHOA); with proteins involved in cellular membrane transport.	[8,9,48]
NSP8	NSP8 interacts with mitochondrial ribosome subunits (MRPS2, MRPS5, MRPS25, MRPS27), with components of the human RNA exosome complex, and with the Signal Recognition Particle (SRP) and can influence mitochondrial translation. NSP8 contains a mitochondrial targeting domain, allowing it to accumulate in mitochondria. Thanks to the autophagy/mitophagy-inducing domain, it increases autophagosome content but blocks their fusion with lysosomes, leading to incomplete mitophagy. NSP8 interacts with various multidrug resistance proteins.	[8,9,48]
NSP10	The NSP10–NSP14 complex functions as an RNase. The NSP10–NSP16 complex changes the viral RNA cap into a structure that imitates host cellular mRNAs. It interacts with NADH4L (NADH-ubiquinone oxidoreductase subunit 4L) and COX II (cytochrome C oxidase subunit II), leading to decreased NADH cytochrome activity and depolarization of the inner mitochondrial membrane. It also interacts with AP2 proteins.	[8,49]
**Structural proteins**		
S protein	S protein causes long-term transcriptional suppression of mitochondrial metabolism genes (including ATP synthase and the CI subunit genes) and increases the expression of genes encoding stress pathway-associated factors and glucose metabolism genes. S protein interacted with monoamine oxidase B (MAO-B) and enhanced its activity, reducing ΔΨm, increasing mt-ROS, and disrupting bioenergetics. It impaired mitophagy by suppressing parkin protein expression in human SH-SY5Y neuroblastoma cells. S protein subunits affected ΔΨm, reduced respiration rate, and increased mitochondrial fission in human lung microvascular endothelial cells (HPMEC). Binding of the S1 subunit to Signal transducer and activator of transcription (STAT) 1 blocks STAT1 phosphorylation by kinases, leading to inhibition of the IFN-I signaling pathway.	[9,50,51]
M protein	The central organizer of virus assembly, it interacts with the S and N proteins, regulating the size and variability of the virion. The M protein interacts with many mitochondrial proteins, including medium-chain acyl-CoA dehydrogenase (ACADM), pitrilizine metallopeptidase 1 (PITRM1), the α- and β-subunits of mitochondrial processing peptidase (PMPCA and PMPCB), coenzyme Q8B (COQ8B), leucyl-tRNA (TARS2) and asparaginyl-tRNA (NARS2) synthase of mitochondria, members of the solute carrier (SLC) family, ATPase Na^+^/K^+^-transporting subunit β1 (ATP1B1), and vacuolar ATPase H^+^-transporting subunit V1 A (ATP6V1A). The M protein prevents the assembly and signaling of MAVS. It also disrupts the movement of IRF3 into the nucleus through KPNA6, which attenuates IFNβ production and transcription of interferon-stimulated genes. The M protein promotes mitochondrial apoptosis by stabilizing the BCL-2 ovarian killer (BOK) protein.	[8,9,50]
E protein	The E protein is a viroporin involved in the release of viral particles from the cell. Together with the M protein, it regulates the intracellular transport and processing of the S protein. It induces VDAC1 overexpression and VDAC1 oligomerization, leading to the rearrangement of interorganellar contact sites, mitochondrial remodeling, mtDNA release, disruption of Ca^2+^ homeostasis, and apoptosis.	[9,52]
N protein	The N protein affects viral replication, transcription, and assembly, along with NSPs. It interacts with the MCL-1 protein and suppresses cell apoptosis; it disrupts the assembly of cyclic AMP-GMP synthase (cGAS) with its cofactor G3BP1, which reduces IFN-I signaling. This counteracts the accumulation of mt-DNA fragments that activate the cGAS pathway. The N protein raises mt-ROS levels and affects ATP production. It attaches to various subunits of CI, enhances CI and CIII activity, and increases mitochondrial gene expression for the ETC complexes. It also connects with PMPCA and PMPCB, helping to stabilize mitochondrial transcription complexes.	[9,29]
**Accessory proteins**		
ORF3a	ORF3a is associated with the creation of DMVs, probably by harnessing the lipid metabolism of the host cell. Depending on the cell type, ORF3a can induce apoptosis and suppress IFN-I signaling by preventing STAT1 activation and its nuclear translocation, leading to increased suppressor of cytokine 1 (SOCS1) signaling, or it increases the transcription of genes associated with innate immune functions, including interferon-regulated genes (*IFI44L*), the Toll-like receptor 5 gene (*TLR5*), genes of the complement and coagulation cascades, *SLC9A9*, *SLC16A7*, *SLC16A9*, and *SLC25A27* transporters, as well as enzymatic regulators of metabolism (e.g., *ADH4* and *ACSL5*). ORF3a causes significant changes in the bile acid metabolism pathway and decreases expression of mTORC1, hypoxia, glycolysis, OXPHOS, or mt-ROS. ORF3a suppressed the expression of genes encoding components CI, III, IV, and V and genes responsible for mitochondrial cristae morphology and function, including optical atrophy protein 1 (*OPA1*), *SAMM50*, *TOMM40*, *MIC60* (*IMMT*), and *MIC13* (*MICOS13*). ORF3a targets USP30, which is a protein that restricts mitophagy and controls the fission and fusion of mitochondria. It promotes mitochondrial fission and alters cristae architecture, which reduces ΔΨm and increases motility in a significant mitochondrial population. ORF3a binds to the NLRP3 inflammasome. ORF3a impacts cell energy use, carbohydrate and cofactor handling, and nucleotide production.	[8,53]
ORF3b	ORF3b interacts with the RIG-I-like receptor (RLR) and inhibits RIG-I- and MAVS-mediated IFN-I induction.	[8]
ORF3c	ORF3c localizes to mitochondria and interacts with MAVS and PGAM5, activating caspase-3 to cleave MAVS, which reduces IFN-β production and suppresses innate immunity. This occurs after RIG-I or MDA5 activation, but before TIR domain-containing adaptor inducing IFN-β (TRIF) or TANK-binding kinase 1 (TBK1) activation.	[9]
ORF6	The ORF6 protein, in conjunction with ORF9b, directly inhibits IFN signaling within the infected cell.	[9]
ORF7a	ORF7a is localized primarily in the ER and Golgi apparatus, and partially in mitochondria. ORF7a binds Bcl-XL, Bcl-2, Bcl-w, Mcl-1, and A1 proteins to induce apoptosis, but does not interact with proapoptotic proteins such as Bax, Bak, Bad, and Bid.	[8]
ORF7b	ORF7b is a multifunctional protein that binds to a number of key host cell proteins, reprogramming their signaling pathways. It interacts with MAVS, suppressing IFN-I production. ORF7b links up with the N protein, which seems to aid in the virus’s assembly.	[54,55]
ORF8	ORF8 joins with ER oxidoreductases to produce mixed disulfide complexes. ORF8 leads to ER stress via the Unfolded Protein Response pathway and aids in viral replication by ORF8 interacting with calnexin.	[56,57]
ORF9b	ORF9b localizes to the outer mitochondrial membrane, induces DMV formation, and, together with NSP4, forms outer mitochondrial membrane macropores. It interacts with mitochondrial proteins such as TOM70, MAVS, and the mitophagy receptor Nip3-like protein X (NIX). ORF9b induces mitochondrial fragmentation, but has also been reported to induce mitochondrial elongation through ubiquitination and degradation of Drp1. ORF9b suppresses IFN-I responses or antagonizes both IFN-I and IFN-III. ORF9b inhibits the interaction between TOM70 and Hsp90, which is required for recruitment of the Hsp90/TBK1/IRF3 complex within mitochondria. ORF9b decreases Stimulator of Interferon Response cGAMP Interactor 1 (STING1) expression. ORF9b alters amino acid and lipid metabolism.	[8,53]
ORF9c	ORF9c interacts with TOM70, MAVS, or NIX, along with NDUFAF1 (which helps build the NADH:ubiquinone oxidoreductase complex) and NDUFAB1 (a subunit of NADH:ubiquinone oxidoreductase). This might change the start of electron flow and ROS production. ORF9c directly inhibits MAVS. ORF9c induces mitochondrial fragmentation through the degradation of Drp1.	[8,53,58]
ORF10	ORF10 accumulates in mitochondria. It interacts with NIX and Microtubule-associated protein 1A/1B-light chain 3 (LC3), triggering mitophagy, resulting in MAVS degradation and suppression of IFN-I responses. ORF10 suppresses innate immunity by disrupting cGAS-STING signaling. ORF10 increases the expression of genes responsible for the ETC and *OPA1*, *SAMM50*, or *TOMM40*, and decreases the expression of *MIC26*, *MIC27*, and especially *ATP5MF*. ORF10 caused mitochondrial fragmentation, but in some cases promoted mitochondrial fusion.	[8,53,59]

**Table 2:** Changes in gene expression during acute infection (COVID-19) and in long COVID: a possible link to some phenotypic manifestations of long COVID.

	COVID-19	Long-COVID		
Sample	OXPHOS Complex Genes [29]	OXPHOS Complex Genes [138]	Mitophagy	Possible Effect
Lung ^1^	↓ C I-V	↑ C I-V	↓ [140,141]	Fibrosis [140]
	↑ mt C	NE		
Nasopharyngeal ^1^	↓ C I-V	↑ C I-V		
	↓ mt C	NE		
Heart ^1^	↓ C I-V	↓ C I-V	↓ [142,143]	myocarditis and heart failure [142]
	↓ mt C	NE		
Kidney ^1^	↓ C I-III, ↑ C V	↑ C I-III		renal fibrosis [144]
	↓mt C	NE		
Liver ^1^	↓ C I-V, ↑ C I, IV, V	↑ C I-V		
	↓ mt C	NE		
Lymph node ^1^	↓ C II, IV, V	↑↓		
	↓mt C	NE		
Brain				cognitive and mental dysfunction
Cerebellum ^2^	↓ C I-V	NE		
	↓ mt C	NE		
Striatum ^2^	↑ C I-V	NE		
	↑ mt C	NE		
Frontal cortex ^1^	NE	↓	↓	
Substantia nigra ^1^	NE	↑		

^1^ SARS-CoV-2–infected human tissues, ^2^ SARS-CoV-2–infected hamster tissues. mt C—mtDNA-encoded OXPHOS complex genes. C—nDNA-encoded OXPHOS complex genes. ↑ up-regulation of gene expression. ↓ down-regulation of gene expression or inhibition of mitophagy. NE not examined.

## Data Availability

No new data were created or analyzed in this study. Data sharing is not applicable to this article.
